# Carboxylesterase 2-Engineered Stem Cell Therapy Shows Superior Efficacy over Cytosine Deaminase in Castration-Resistant Prostate Cancer

**DOI:** 10.3390/biomedicines14030681

**Published:** 2026-03-16

**Authors:** Jae Heon Kim, Miho Song, Sang Hun Lee, Yun Seob Song

**Affiliations:** 1 Department of Urology, Soonchunhyang University School of Medicine, Seoul 140-743, Republic of Korea; piacekjh@hanmail.net (J.H.K.); miho@schmc.ac.kr (M.S.); 2 Program in Biomedical Sciences & Engineering, Department of Biomedical Sciences, College of Medicine, Inha University, Incheon 22332, Republic of Korea

**Keywords:** castration-resistant prostate cancer, enzyme–prodrug therapy, adipose-derived stem cells, cytosine deaminase, carboxylesterase 2, gene therapy

## Abstract

**Purpose**: Castration-resistant prostate cancer (CRPC) responds poorly to conventional chemotherapy. We evaluated a cell-based enzyme–prodrug therapy using adipose-derived stem cells (ADSCs) engineered to express cytosine deaminase (CD) or carboxylesterase 2 (CE2), paired with their respective prodrugs 5-fluorocytosine (5-FC) or irinotecan (CPT-11), to compare their antitumor efficacy. **Materials and Methods**: Human telomerase reverse transcriptase (hTERT)-immortalized ADSCs were transduced with CD or CE2, and transgene expression and stem cell phenotype were confirmed. CD expression was verified at the transcript level and by functional 5-FC-to-5-fluorouracil (5-FU) conversion, whereas CE2 expression was verified by transcript analysis and immunoblotting. Tumor tropism toward PC3 prostate cancer cells was tested using migration assays and analysis of chemoattractant ligand/receptor expression. Prodrug-induced self-killing and bystander tumor cell killing were assessed through viability assays and co-culture with PC3 cells. For the CE2/CPT-11 system, SN-38 was not directly quantified; functional activity was inferred from prodrug-dependent cytotoxicity and in vivo efficacy. In vivo efficacy was evaluated in nude mice with PC3 tumors treated systemically with engineered ADSCs plus prodrug. **Results**: CD- and CE2-expressing ADSCs were successfully established and retained mesenchymal stem cell (MSC) characteristics. Both cell types exhibited significant migration toward PC3 cells. The CE2/CPT-11 system produced stronger prodrug-mediated cytotoxicity than CD/5-FC, with CE2-modified ADSCs showing higher sensitivity to CPT-11 and inducing greater apoptosis in co-cultured PC3 cells. In vivo, both treatments suppressed tumor growth, but CE2/CPT-11 achieved greater inhibition (tumor volume ~26% of control vs. ~32% for CD/5-FC at day 14). No overt clinical toxicity was observed based on body weight and daily clinical monitoring; however, hematology/serum chemistry were not assessed. **Conclusions**: Engineered ADSCs home to CRPC tumors and enable local prodrug activation, producing significant antitumor effects. Within the constraints of our in vitro assays and subcutaneous xenograft model, CE2/CPT-11 demonstrated stronger efficacy outcomes than CD/5-FC. Mechanistic attribution to intratumoral SN-38 exposure should be confirmed by direct metabolite measurements in future studies.

## 1. Introduction

Prostate cancer (PCa) is a major male malignancy and the second leading cause of cancer-related deaths worldwide [1]. Treating castration-resistant prostate cancer (CRPC), which no longer responds to androgen deprivation therapy, remains difficult, as available treatments—including chemotherapy—provide limited benefit and often cause significant toxicity [1]. This highlights the need for more effective and safer therapeutic strategies.

Cell-based gene therapy offers a targeted approach that may reduce systemic side effects [2,3]. In enzyme/prodrug therapy (EPT), engineered cells express an enzyme that converts a systemically delivered, non-toxic prodrug into an active chemotherapeutic agent specifically at the tumor site [2,3]. Adipose-derived stem cells (ADSCs) are well suited for EPT due to their accessibility, efficient gene modification, and intrinsic tumor-homing capacity [4,5,6,7]. Autologous ADSCs also minimize immune rejection and allow repeated administration.

Two widely studied EPT platforms are the cytosine deaminase/5-fluorocytosine (CD/5-FC) system and the carboxylesterase/irinotecan (CE/CPT-11) system [2,3]. CD converts 5-FC into 5-fluorouracil (5-FU), which exerts strong bystander cytotoxicity; ADSCs expressing CD enhance 5-FC-mediated tumor inhibition in CRPC models [8]. CE2 converts CPT-11 into SN-38, a topoisomerase inhibitor 100–1000 times more potent than CPT-11 [9]. Because human CPT-11 activation depends primarily on hepatic and intestinal CE2—and blood lacks significant esterase activity [10,11]—efficient local conversion is crucial. Human CE2 displays ~100-fold higher catalytic efficiency than CE1 [12], while rabbit CE2 is ~100-fold more active still and has shown superior antitumor effects in preclinical studies [13]. ADSCs engineered with CE2 have previously demonstrated enhanced CPT-11-mediated tumor suppression in CRPC [14,15]. Compared with 5-FU, which mainly targets rapidly dividing cells and may generate toxic metabolites [3,9,13], SN-38 provides potent DNA-damaging activity and can diffuse effectively within tumor tissue [3,13], suggesting potential advantages for solid tumors like CRPC.

In this study, we engineered hTERT-immortalized ADSCs to express either bacterial CD or rabbit CE2, generating hTERT-ADSC.CD and hTERT-ADSC.CE2 therapeutic lines. The hTERT-immortalized ADSC platform provides a stable and expandable cell source without altering key ADSC properties [16]. We compared the tumor tropism, prodrug-mediated cytotoxicity, and in vivo antitumor efficacy of the CD/5-FC and CE2/CPT-11 EPT systems in a CRPC model. We hypothesized that the CE2/CPT-11 system would show equal or superior antitumor activity, supporting its potential as an ADSC-based gene therapy for advanced prostate cancer.

## 2. Materials and Methods

### 2.1. Cell Lines and Culture

An immortalized human adipose-derived mesenchymal stem cell line (ASC52-Telo; hTERT-ADSC, ATCC, Manassas, VA, USA) served as the therapeutic cell platform. Cells were maintained in DMEM supplemented with 10% heat-inactivated FBS (Gibco, Thermo Fisher Scientific, Waltham, MA, USA), L-glutamine (Gibco, Thermo Fisher Scientific, Waltham, MA, USA), and penicillin/streptomycin (Gibco, Thermo Fisher Scientific, Waltham, MA, USA) at 37 °C in 5% CO_2_. The human prostate cancer line PC3 (androgen-independent CRPC model; Korean Cell Line Bank, Seoul, Republic of Korea) was cultured under identical conditions. All lines were passaged using trypsinization.

This study was conducted in accordance with the guidelines of the Institutional Animal Care and Use Committee (IACUC). The animal protocol was reviewed and approved by the Institutional Animal Research Ethics Committee (IRB) of Soonchunhyang University Seoul Hospital, Bucheon Hospital, Cheonan Hospital, and Gumi Hospital under the approval code 2019-4, and the study was approved on 5 March 2019.

### 2.2. Generation of CD- and CE2-Expressing ADSCs

hTERT-ADSCs were transduced with lentiviral vectors encoding either *Escherichia coli* cytosine deaminase (CD) or rabbit liver carboxylesterase-2 (CE2) under a ubiquitin promoter with puromycin selection. Viral particles were generated in 293T cells, and early-passage ADSCs were infected with viral supernatant plus polybrene (Sigma-Aldrich, St. Louis, MO, USA). Stable populations were obtained after 2-week puromycin (Sigma-Aldrich, St. Louis, MO, USA) selection and designated hTERT-ADSC.CD and hTERT-ADSC.CE2; unmodified ADSCs were used as controls.

### 2.3. Verification of Transgene Expression

Transgene integration and mRNA expression were confirmed by reverse transcription–polymerase chain reaction (RT-PCR) and quantitative polymerase chain reaction (qPCR) using gene-specific primers, with *GAPDH* and *ACTB* (*β-actin*) as internal controls. The primer sequences used for RT-PCR and qPCR are listed in Table 1. Protein expression was assessed by Western blot using antibodies against CE2, stemness markers (OCT4, NANOG, SOX2), and β-actin. Because the CD protein was not verified by antibody-based immunoblotting in this study, CD confirmation is reported as transcript-level expression with functional enzyme activity evidence rather than antibody-confirmed protein detection.

### 2.4. Stem Cell Phenotype Assessment

To determine whether genetic modification altered ADSC characteristics, flow cytometry was performed for mesenchymal markers (CD29, CD90, CD105) and hematopoietic markers (CD34, CD45). Stemness markers were confirmed by Western blot. Morphology was evaluated microscopically, and all engineered lines retained the fibroblast-like appearance of parental hTERT-ADSCs.

### 2.5. Enzyme Activity Assays

CD enzymatic function was tested using a 5-FC-to-5-FU conversion assay with thin-layer chromatography. hTERT-ADSC.CD lysates converted >90% of 5-FC to 5-FU under assay conditions, confirming robust activity. For the CE2/CPT-11 system, direct SN-38 quantification (e.g., HPLC or LC–MS/MS) was not performed in this study; therefore, CE2 functional activity is presented as prodrug-dependent cytotoxicity and in vivo efficacy rather than direct metabolite measurement.

### 2.6. Tumor Tropism (Migration) Assay

Tumor-directed motility was evaluated using Matrigel-coated transwell inserts (8 µm pores; Corning, Corning, NY, USA), i.e., a transwell invasion-format assay. The lower chamber contained either medium alone, WPMY-1 cells, or PC3 cells. ADSCs (unmodified, CD-, or CE2-expressing) were plated in the upper chamber and allowed to migrate for 48 h. Migrated cells on the underside of the membrane were stained and quantified microscopically. Quantification was performed by counting stained cells in five randomly selected microscopic fields per insert, with three inserts per condition. Analysis was performed blinded to group allocation. Representative images include scale bars.

### 2.7. Chemoattractant Ligand/Receptor Profiling

RT-PCR was used to examine expression of *SCF, SDF-1, VEGF*, and their receptors (*c-kit, CXCR4, VEGFR1-3*) in control vs. engineered ADSCs to assess potential changes in tumor-homing pathways.

### 2.8. Prodrug Cytotoxicity (Suicide) Assay

Engineered ADSCs were exposed to increasing concentrations of 5-FC (CD cells) or CPT-11 (CE2 cells) for 72 h. Cell viability was measured by MTT assay and expressed relative to untreated controls. Unmodified hTERT-ADSCs were treated in parallel with 5-FC and CPT-11 to serve as prodrug-only controls.

### 2.9. Tumor Cell Apoptosis Co-Culture Assay

PC3 cells were co-cultured with hTERT-ADSC.CD or hTERT-ADSC.CE2 in the presence of prodrug for 48–72 h, and PC3 viability/response was assessed as a readout of bystander effects. Control co-cultures included (i) PC3 alone ± prodrug and (ii) PC3 co-cultured, with unmodified hTERT-ADSCs ± prodrug to distinguish enzyme-dependent bystander effects from non-specific prodrug effects.

### 2.10. In Vivo CRPC Xenograft Model

Male BALB/c nude mice (6–8 weeks) were injected subcutaneously with PC3 cells to establish tumors. Once tumors became palpable, mice were assigned to three groups: PBS control, CD/5-FC therapy, or CE2/CPT-11 therapy. Group allocation was randomized, and tumor measurements were performed blinded when feasible. Each treatment group received intracardiac injection of 1 × 10^6^ engineered ADSCs on Day 7. Prodrug dosing was initiated on Day 21 (14 days after ADSC administration). CPT-11 was administered via intraperitoneal injection at 1.7 mg/kg per dose following a 5-days-on/2-days-off schedule for two cycles. The 5-FC was administered via intraperitoneal injection at 500 μg/kg/day (once daily) using the same schedule. Tumors were measured weekly, and tumor volume was calculated as V = (length × width^2^)/2.

Toxicity monitoring included body-weight tracking and daily clinical observation for diarrhea, grooming/activity changes, and signs of distress; humane endpoints were predefined.

### 2.11. Statistical Analysis

For in vitro experiments, *n* denotes independent biological replicates (experiments independently repeated ≥3 times unless otherwise stated), and data are presented as mean ± SD. For in vivo studies, *n* denotes the number of mice per group, and data are presented as mean ± SEM. Group comparisons over time were analyzed using two-way ANOVA with a post hoc multiple comparisons test (e.g., Tukey). Exact tests and comparison pairs are stated in each figure legend. Statistical significance was set at *p* < 0.05. All statistical analyses were performed using GraphPad Prism software (version 9.0, GraphPad Software, Boston, MA, USA).

## 3. Results

### 3.1. Establishment of CD- and CE2-Expressing ADSCs

**Figure 1:**
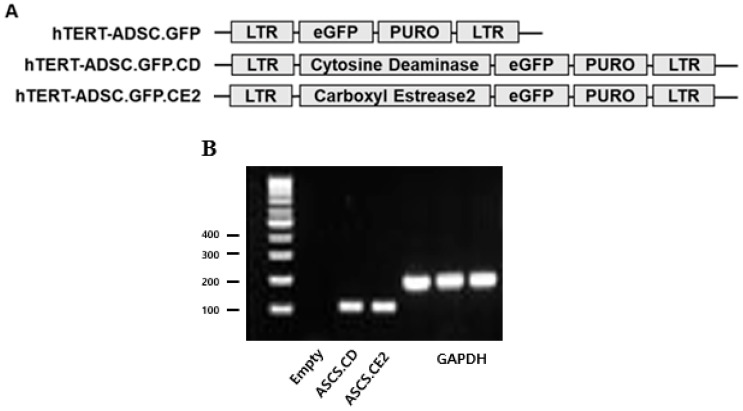
Generation of CD- and CE2-overexpressing human telomerase reverse transcriptase (hTERT)-immortalized adipose-derived stem cells (hTERT-ADSC.CD and hTERT-ADSC.CE2). (**A**) hTERT-hADSC, hTERT-hADSC.CD, and hTERT-hADSC.CE2 were generated via lentiviral transduction of GFP, CD, and CE2 gene using CLV-Ubic vector. (**B**) The expression of CD and CE2 was confirmed by RT-PCR. ADCS = hTERT-hADSC, CD = cytosine deaminase, CE2 = carboxyl esterase 2, RT-PCR = reverse transcription–polymerase chain reaction.

hTERT-immortalized ADSCs were successfully engineered to express CD or CE2 via lentiviral transduction, followed by puromycin selection. RT-PCR verified stable integration, showing clear CD and CE2 bands in the corresponding engineered lines, with no CD signal in hTERT-ADSC.CE2 (Figure 1).

### 3.2. Functional Enzyme Activity

**Figure 2:**
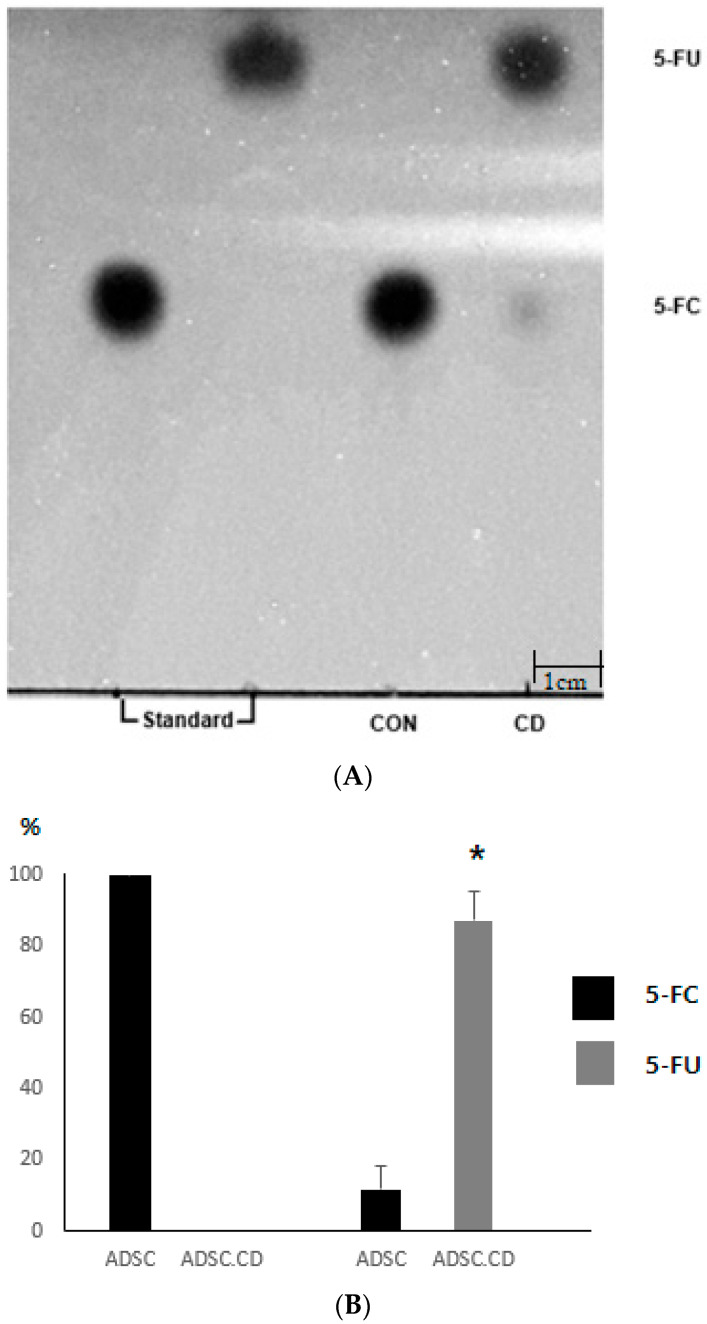
Thin-layer chromatography. (**A**) In vitro cytosine deaminase (CD) activity was determined by thin-layer chromatography (TLC) to measure the conversion of 5-fluorocytosine (5-FC) to 5-fluorouracil (5-FU) by CD present in the lysates. 5-FC and 5-FU standard, control (hTERT-hADSC) and hTERT-hADSC.CD are 100 mmol each, respectively. Significant changes were not observed after 5-FC incubation with hTERT-hADSC; however, the conversion of 5-FC to 5-FU took place in hTERT-hADSC.CD. Scale bar = 1 cm. The TLC plate image represents a cropped region (6 × 9 cm) of the original plate, and the scale bar indicates the physical distance on the chromatographic plate. (**B**) Quantitative expression of 5-FC or 5-FU in hTERT-ADSC, hTERT-ADSC.CD = hTERT-hADSC, CD. * *p* < 0.05 vs. control.

Engineered cells demonstrated robust prodrug-converting activity. TLC of hTERT-ADSC.CD lysates showed strong conversion of 5-FC to 5-FU (>90%), whereas control ADSCs produced no detectable 5-FU. These data confirm functional CD enzyme expression (Figure 2).

### 3.3. Tumor-Tropic Migration

**Figure 3:**
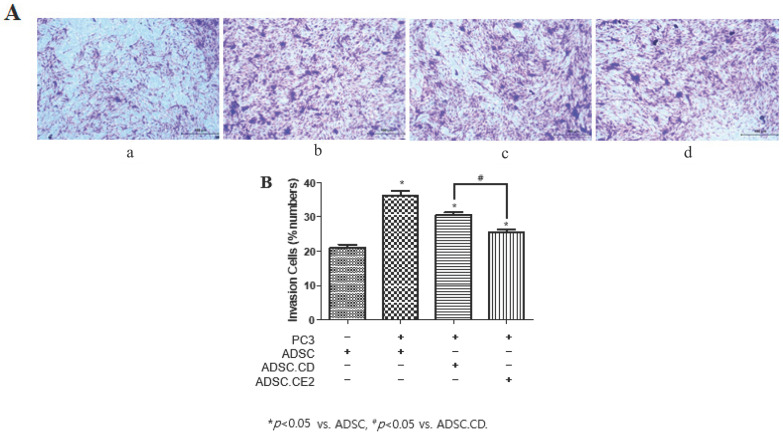
Tumor-directed migration of engineered hTERT-ADSCs toward PC3 prostate cancer cells. (**A**) Representative images of migrated cells in Matrigel-coated transwell inserts (8 μm pore size). Cells that migrated to the lower surface of the membrane were fixed and stained with crystal violet, resulting in purple/blue staining of migrated cells. Darker staining indicates increased numbers of migrated cells. a. Migration toward control medium. b. Migration toward WPMY-1 stromal cells. c. Migration toward PC3 prostate cancer cells. d. Migration toward PC3 cells. Scale bar = 100 μm. (**B**) Quantification of migrated cells per microscopic field. Cells were counted in five randomly selected microscopic fields per insert, using three inserts per experimental condition. Quantification was performed blinded to group allocation. Data are presented as mean ± SD from three independent experiments. Statistical analysis was performed using one-way ANOVA followed by Tukey’s multiple comparisons test.

Both engineered ADSC types retained potent tumor-homing capacity. In transwell assays, hTERT-ADSC.CD and hTERT-ADSC.CE2 demonstrated significantly increased migration toward PC3 cells compared with medium alone or WPMY-1 cells (*p* < 0.05). Minimal migration occurred in control conditions, whereas PC3-conditioned wells elicited strong chemotaxis (Figure 3A). Quantification showed significantly higher migration in both engineered groups and a modest difference between CD and CE2 lines (*p* < 0.05) (Figure 3B). Genetic modification did not impair homing ability.

### 3.4. Chemoattractant Factor Expression

**Figure 4:**
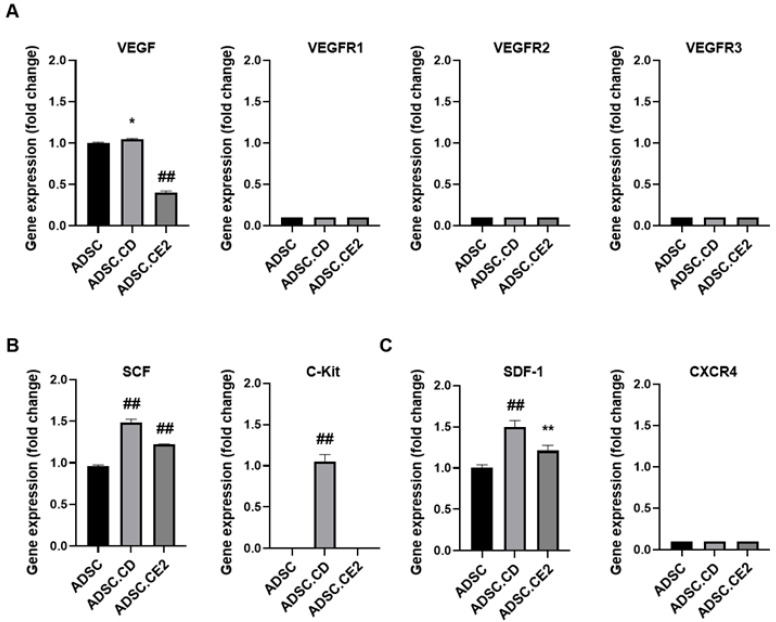
Relative mRNA expression of chemoattractant ligand and receptor genes in engineered hTERT-ADSCs. Expression levels of chemoattractant ligands and receptors were analyzed by real-time PCR in parental hTERT-ADSCs and genetically modified ADSCs. (**A**) Relative mRNA expression of SCF, SDF-1, and VEGF in hTERT-ADSC.CD cells compared with parental hTERT-ADSCs. (**B**) Relative mRNA expression of SCF, SDF-1, and VEGF in hTERT-ADSC.CE2 cells compared with parental hTERT-ADSCs. (**C**) Relative mRNA expression of the receptor gene KIT in engineered hTERT-ADSCs. Compared with parental hTERT-ADSCs, the expression levels of VEGF, SCF, SDF-1, and KIT were significantly increased in hTERT-ADSC.CD cells, whereas VEGF expression was decreased and SCF and SDF-1 levels were reduced in hTERT-ADSC.CE2 cells. Data are presented as mean ± SD from three independent experiments. * *p* < 0.05, ** *p* < 0.01 vs. hTERT-ADSC (control); ## *p* < 0.01 vs. hTERT-ADSC.CD. Abbreviations: ADSC, adipose-derived stem cell; SCF, stem cell factor; SDF-1, stromal cell-derived factor-1; VEGF, vascular endothelial growth factor; KIT, KIT proto-oncogene receptor tyrosine kinase; VEGFR, vascular endothelial growth factor receptor.

RT-PCR profiling showed that parental ADSCs express several tumor-homing ligands and receptors. CD modification modestly increased VEGF, SCF, SDF-1, and their receptors (VEGFRs, c-kit, CXCR4), suggesting enhanced autocrine signaling (*p* < 0.05 vs. control). CE2-modified ADSCs showed slight reductions in the same factors (*p* < 0.05), yet still migrated efficiently, implying that baseline receptor expression or additional PC3-secreted cues supported migration. Overall, both cell types retained key chemotactic signaling components (Figure 4).

### 3.5. In Vitro Suicide Effect and Cytotoxic Potency

**Figure 5:**
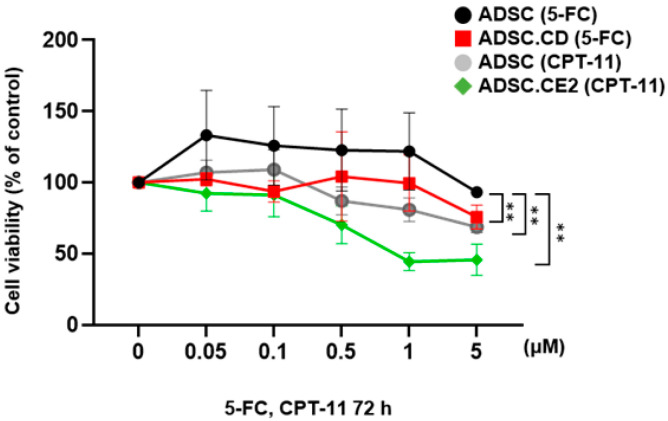
Prodrug-dependent cytotoxic response of engineered hTERT-ADSCs following treatment with 5-FC or CPT-11. Engineered ADSCs were exposed to increasing concentrations of the corresponding prodrugs for 72 h. hTERT-ADSC.CD cells were treated with 5-fluorocytosine (5-FC), whereas hTERT-ADSC.CE2 cells were treated with irinotecan (CPT-11). Unmodified hTERT-ADSCs treated with prodrugs served as prodrug-only controls to exclude intrinsic cytotoxicity unrelated to enzyme expression. Cell viability was measured and expressed as a percentage of untreated control cells. Data are presented as mean ± SD from three independent experiments. Statistical analysis was performed using two-way ANOVA followed by Tukey’s multiple comparisons test. Significance symbols indicate the following comparisons: ** *p* < 0.01 vs. hTERT-ADSC control.

Prodrug exposure confirmed functional enzyme-mediated self-killing. Unmodified hTERT-ADSCs treated with 5-FC or CPT-11 in parallel maintained viability across tested doses, supporting that observed killing in engineered lines was enzyme-dependent under our assay conditions. hTERT-ADSC.CD showed reduced viability at higher 5-FC concentrations, whereas hTERT-ADSC.CE2 showed prodrug-dependent cytotoxicity at lower CPT-11 concentrations. However, due to differences in intrinsic prodrug pharmacology and the absence of active-metabolite (SN-38) quantification, these dose–response thresholds should not be interpreted as a direct comparison of enzyme catalytic efficiency (Figure 5).

### 3.6. In Vivo Tumor Suppression

**Figure 6:**
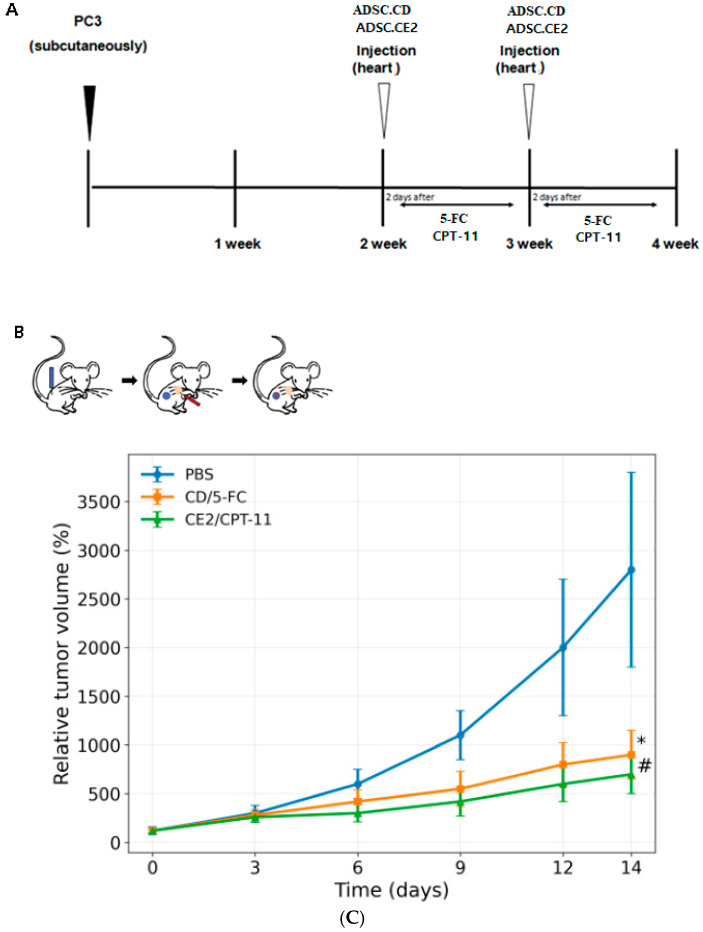
In vivo efficacy and observational tolerability in a subcutaneous PC3 xenograft model. (**A**) Study timeline and dosing schedule: engineered ADSCs (1 × 10^6^ cells, intracardiac injection, Day 7); prodrug dosing initiated on Day 21 using a 5-days-on/2-days-off × 2 cycles schedule. CPT-11 was administered 1.7 mg/kg i.p. per dose; 5-FC was administered 500 μg/kg/day i.p. (**B**) Schematic of tumor establishment and systemic ADSC delivery with tumor-directed homing concept. (**C**) Tumor growth curves; tumor volume calculated as V = (length × width^2^)/2. Group size n = 5 mice per arm. Data are mean ± SEM. Statistical analysis: two-way ANOVA with post hoc multiple comparisons. * *p* < 0.05 vs. PBS; # *p* < 0.05 CD/5-FC vs. CE2/CPT-11.

Systemic administration of engineered ADSCs combined with prodrug treatment significantly reduced PC3 tumor growth in mice. Control tumors expanded ~27.9-fold over 14 days. CD/5-FC therapy limited growth to ~8.9-fold, while CE2/CPT-11 further reduced growth to ~7.2-fold (Figure 6). Both treatments significantly decreased tumor volume versus control (*p* < 0.05). CE2/CPT-11 produced the strongest suppression, yielding tumors ~19% smaller than those in the CD/5-FC group by the end of the study (*p* < 0.05) (Figure 6C).

## 4. Discussion

This study provides a direct comparison of two ADSC-based enzyme/prodrug therapies for CRPC—CD/5-FC versus CE2/CPT-11—and demonstrates that both approaches are effective, with CE2/CPT-11 showing superior antitumor activity. Our results reinforce the feasibility of using ADSCs as gene-modified carriers for EPT and highlight the advantage of leveraging their intrinsic tumor tropism [5,6,7]. These findings support the concept that mesenchymal stem cell-based enzyme/prodrug systems can function as targeted delivery platforms capable of concentrating therapeutic activity within tumor tissues while potentially limiting systemic exposure [2,3].

Both CD- and CE2-expressing hTERT-ADSCs migrated efficiently toward PC3 cells, consistent with functional *SDF-1/CXCR4, SCF/c-kit*, and *VEGF/VEGFR* signaling. This supports the concept that ADSCs can deliver therapeutic enzymes directly to tumors while minimizing systemic exposure. Importantly, the genetic modification required for enzyme expression did not appear to impair the intrinsic tumor-tropic behavior of ADSCs, suggesting that therapeutic engineering can be achieved without compromising key biological properties of ADSCs [5,6,7].

The CD/5-FC system served as a benchmark, and our findings align with previous work showing significant tumor inhibition using CD-engineered ADSCs [8]. However, CD/5-FC therapy is constrained by the biology of 5-FU, which primarily targets proliferating cells and generates metabolites toxic to normal dividing tissues. Moreover, the cytotoxic activity of 5-FU largely depends on tumor cell proliferation, which may limit its therapeutic impact in heterogeneous tumors containing slow-cycling or quiescent cancer cell populations. Its limited activity in poorly perfused or slow-growing tumor regions may further restrict efficacy.

In contrast, the CE2/CPT-11 system offers pharmacological advantages. CPT-11 is clinically used, and its conversion to SN-38—a highly potent topoisomerase I inhibitor—typically occurs in the liver, contributing to systemic toxicity [9,10,11]. By engineering ADSCs to locally express CE2 within the tumor microenvironment, the conversion of CPT-11 to SN-38 is expected to occur preferentially at the tumor site, potentially increasing intratumoral drug exposure while reducing systemic activation [12,13]. The CE2-expressing ADSCs displayed strong “suicide” sensitivity to low CPT-11 concentrations and induced pronounced killing of surrounding cancer cells.

Consistent with this mechanism, the CE2/CPT-11 group showed greater cytotoxic responses *in vitro* and stronger tumor suppression *in vivo* than CD/5-FC. Although SN-38 concentrations were not directly measured in this study, the enhanced cytotoxicity observed *in vitro* and the stronger tumor growth inhibition *in vivo* are consistent with effective local activation of CPT-11 by CE2-expressing ADSCs. Importantly, the present findings reflect functional tumor cell killing rather than direct mechanistic confirmation of apoptosis induction, as quantitative analyses of apoptosis-related markers were not performed in this study. This improved efficacy likely reflects (1) the markedly higher catalytic efficiency of rabbit CE2 [12,13], (2) the greater potency and broader cell-cycle activity of SN-38 relative to 5-FU [9], and (3) more favorable diffusion properties that enhance bystander killing.

We observed differential expression of chemoattractant factors between CD- and CE2-modified ADSCs, with CD-engineered cells showing modest upregulation of VEGF, SCF, and SDF-1, whereas CE2 cells showed slight reductions. Despite this, both migrated similarly toward tumor cues, suggesting that baseline receptor expression or additional tumor-derived factors were sufficient. These findings indicate that modest transcriptional alterations induced by genetic modification may not necessarily translate into functional impairment of tumor-directed migration. The functional relevance of the expression differences remains unclear, but it did not impact therapeutic performance.

Safety considerations also support the EPT approach. Both engineered ADSC types were eliminated following prodrug treatment due to the suicide effect, reducing the risk of long-term persistence or transformation [16]. The hTERT-immortalized ADSC line provided a stable, non-tumorigenic, and expandable cell source, consistent with previous reports showing that hTERT expression does not induce malignant transformation [16]. Nevertheless, long-term biodistribution and persistence of engineered ADSCs should be investigated in future translational studies to ensure safety in potential clinical applications.

Several limitations of this study should also be acknowledged. First, SN-38 production was inferred indirectly through functional cytotoxicity rather than direct metabolite quantification, and, therefore, the precise intratumoral pharmacokinetics remain to be determined. Second, the in vivo experiments were conducted using a subcutaneous xenograft model, which may not fully replicate the complex tumor microenvironment observed in advanced or metastatic CRPC. Third, systemic toxicity evaluation was limited to body weight and clinical observation, and comprehensive hematologic or biochemical analyses were not performed.

While CE2/CPT-11 demonstrated strong therapeutic potential, translation will require addressing immunogenicity concerns, as rabbit CE2 is a foreign protein. Human CE1/CE2 variants with enhanced activity may represent future alternatives [3]. Further optimization of enzyme selection, delivery strategy, and prodrug dosing will be necessary to improve the therapeutic index and facilitate future clinical translation.

Overall, our findings support ADSC-based EPT as a promising strategy for CRPC. Autologous ADSCs could be isolated, modified to express an optimized enzyme, and reintroduced alongside a systemic prodrug to achieve high intratumoral drug concentrations and overcome tumor heterogeneity. Taken together, these results suggest that CE2-engineered ADSCs combined with CPT-11 represent a potentially effective cell-mediated prodrug therapy approach for advanced prostate cancer, although further mechanistic and translational studies will be required to validate this strategy.

## 5. Conclusions

In this study, we show that human adipose-derived stem cells engineered to express therapeutic enzymes can effectively deliver and activate prodrugs within prostate tumors. Both CD/5-FC and CE2/CPT-11 gene-modified ADSCs retained strong tumor-homing ability and exerted significant antitumor effects in CRPC models. Importantly, the CE2/CPT-11 system produced markedly stronger tumor suppression than the conventional CD/5-FC strategy, highlighting its superior therapeutic potential. These findings support ADSC-based CE2/CPT-11 therapy as a promising, targeted approach for castration-resistant prostate cancer. Given their intrinsic tumor tropism and feasibility for autologous application, ADSCs may enable localized chemotherapy with reduced systemic toxicity. Future efforts should aim to optimize delivery, ensure safety, and advance this platform toward clinical translation as a novel cell-mediated gene therapy for advanced prostate cancer.

## Figures and Tables

**Table 1:** Primer sequences used for RT-PCR/qPCR.

Gene	Forward Primer (5′ → 3′)	Reverse Primer (5′ → 3′)	Size (bp)
*CD*	GCGCGAGTCACCGCCAGCCACACCACGGC	GTTTGTAATCGATGGCTTCTGGCTGC	559
*CE2*	CCATTGGGATGAAAGGAAGA	AGAAAAGGAGGGAGCAGAGG	200
*SCF*	ACTTGGATTCTCACTTGCATTT	CTTTCTCAGGACTTAATGTTGAAG	505
*c-kit*	GCCCACAATAGATTGGTATTT	AGCATCTTTACAGCGACAGTC	332
*SDF-1*	ATGAACGCCAAGGTCGTGGTC	GGCTGTTGTGCTTACTTGTTT	200
*CXCR4*	CTCTCCAAAGGAAAGCGAGGTGGACAT	AGACTGTACACTGTAGGTGCTGAAATCA	733
*VEGF*	AAGCCATCCTGTGTGCCCCTGATG	GCTCCTTCCTCCTGCCCGGCTCAC	541
*VEGFR1*	GCAAGGTGTGACTTTTGTTC	AGGATTTCTTCCCCTGTGTA	512
*VEGFR2*	ACGCTGACATGTACGGTCTAT	GCCAAGCTTGTACCATGTGCG	438
*VEGFR3*	AGCCATTCATCAACAAGCCT	GGCAACAGCTGGATGTCATA	298
*β-actin*	GCACCACACCTTCTACAATG	TGCTTGCTGATCCACATCTG	619
*GAPDH*	CATGACCACAGTCCATGCCATCACT	TGAGGTCCACCACCCTGTTGCTGTA	450

## Data Availability

The data presented in this study are available on request from the corresponding authors due to ethical restrictions.

## Abbreviations

The following abbreviations are used in this manuscript:

CRPC	castration-resistant prostate cancer
ADSCs	adipose-derived stem cells
CD	cytosine deaminase
CE2	carboxylesterase 2
5-FC	5-fluorocytosine
CPT-11	irinotecan
EPT	enzyme/prodrug therapy
5-FU	5-fluorouracil
